# Multiple system atrophy—cerebellar type: Diagnostic challenge in resource‐limited settings case report

**DOI:** 10.1002/ccr3.9142

**Published:** 2024-07-03

**Authors:** Gebeyehu Tessema Azibte, Bereket Abraha Molla, Sebhatleab Teju Mulate, Selam Kifelew Melkamu, Zekarias Seifu Ayalew

**Affiliations:** ^1^ Department of Internal Medicine Addis Ababa University College of Medicine and Health Sciences Addis Ababa Ethiopia; ^2^ Department of Neurology Addis Ababa University College of Medicine and Health Sciences Addis Ababa Ethiopia

**Keywords:** multiple system atrophy, neurodegenerative disorder, synucleinopathies, tremor

## Abstract

**Key Clinical Message:**

This case report highlights the challenges of diagnosing MSA‐C in resource‐limited settings. MRI findings like the “hot cross bun” sign can be supportive, but the unavailability of advanced tools like seed amplification assay may delay diagnosis. Early diagnosis is crucial for proper symptom management.

**Abstract:**

Multiple system atrophy is a rare neurodegenerative disorder affecting the pyramidal, autonomic, nigrostriatal, and cerebellar tracts. Multisystem atrophy should be considered in adults with progressive motor or autonomic dysfunctions. Clinical manifestations vary depending on the system, including bradykinesia, tremor, rigidity, cerebellar ataxia, and autonomic failure. Depending on the initial predominant manifestation, multisystem atrophy is classified as Parkinsonian (MSA‐P) and cerebellar (MSA‐C). Our patient presented with progressive loss of balance, rigidity, slurred speech, choking episodes, and loss of morning tumescence for 4 years, suggesting autonomic and cerebellar involvement. He was diagnosed with MSA after 4 years of initial presentation with combinations of magnetic resonant imaging findings and clinical manifestations. Diagnosing multiple system atrophy in such resource‐limited areas is challenging. The unavailability of seed application tests and biomarkers significantly affected the delayed diagnosis.

## 1 INTRODUCTION

Multiple system atrophy (MSA) is a neurodegenerative disorder characterized by the intracellular accumulation of Lewy bodies and Lewy neurites in neurons and glial cytoplasmic inclusions oligodendrocytes, consisting primarily of alpha‐synuclein.^1^ Accumulation of alpha‐synuclein is a common feature of synucleinopathies such as Parkinson disease and dementia with Lewy bodies. A conclusive diagnosis of MSA can only be reached after a postmortem examination of glial cytoplasmic inclusions. However, a probable diagnosis of MSA can be established clinically when a characteristic Parkinsonian syndrome with minimal response to levodopa treatment (MSA‐P) or a cerebellar syndrome (MSA‐C) co‐occurs with autonomic dysfunction.^1^

T1 and T2 weighted imaging play a significant role in diagnosing MSA. This approach has identified several “classical” markers, with the “hot cross‐bun” sign being the most prominent. This distinctive pattern reflects degeneration in the pons and pontocerebellar fibers, while the corticospinal tract remains relatively unaffected. Even though this sign has high specificity (97%), it is less sensitive (50%).^2^ Other MRI findings include the ‘putaminal rim’ sign‐ a hyperintense rim of the putamen on T2–T2‐weighted imaging, hyperintensity of the middle cerebellar peduncle, and atrophy of the cerebellum and brainstem.^1^ Real‐time quaking‐induced conversion (RT‐ QuIC) and protein misfolding cyclic amplification (PMCA) have been developed to detect misfolded alpha‐synuclein in cerebrospinal fluid in synucleinopathies. In a systematic review and meta‐analysis by A. Grossauer et al. to evaluate the sensitivity and specificity of αSyn seed amplification assays (αSyn‐SAAs), the pooled sensitivity and specificity were 0.57 (95% CI, 0.26–0.83) and 0.96 (95% CI, 0.91–0.99), respectively, the pooled sensitivity to differentiate MSA from non‐synucleinopathies was 0.30 (95% CI, 0.11–0.59) when only RT‐QuIC seeding method was used. The pooled sensitivity and specificity for differentiating MSA from other synucleinopathies were 0.18 (95% CI, 0.08–0.37) and 0.90 (95% CI, 0.70–0.97), respectively.^3^ This study demonstrated that CSF‐ α‐synuclein RT‐QuIC and PMCA have high diagnostic value for diagnosing synucleinopathies. Measuring biomarkers in speculative CNS‐enriched extracellular vesicles is becoming famous for diagnosing synucleinopathies, but their accuracy in diagnosing MSA is unreliable. A comprehensive systematic review and meta‐analysis by H. B. Taha et al. revealed that biomarkers in speculative CNS‐enriched EVs have lesser accuracy and high heterogeneity for diagnosing MSA.^4^ This study shows that the high level of heterogeneity and absence of standard pre‐analytical factors for the sensitivity of these biomarkers hampers their value in diagnosing synucleinopathies.

The age of onset of MSA (multiple system atrophy) varies across studies. According to Köllensperger et al., from 437 patients recruited from 19 European MSA study group centers, the mean age of onset was 57.8 years. Parkinsonian type (MSA‐P) was reported in 68%, and cerebellar type (MSA‐C) in 32% of patients.^5^ In a systematic review by Ben‐Shlomo et al. of 433 pathologically confirmed MSA cases, the mean age of onset was 54.2 years (ranges 31–78), and survival was 6.2 years.^6^ Based on the initial predominant clinical manifestation, MSA is classified as Parkinsonian‐type (MSA‐P) characterized by akinesia/bradykinesia, rigidity, postural instability, cerebellar type (MSA‐C) manifesting as gait ataxia, limb ataxia, dysarthria or mixed MSA with clinical features of both parkinsonian and cerebellar types. Two‐thirds of patients have action or postural tremors, called mini poly‐myoclonus.^7^ Dysautonomia, such as urinary incontinence, erectile dysfunction, and orthostatic hypotension, are features of Parkinsonian‐type and cerebellar‐type MSA.^8^ Sleep abnormalities are also common in multisystem atrophy. In a retrospective analysis of 45 patients with MSA performed by Rekik S et al., 62.2% had sleep and breathing abnormalities.^9^ cognitive function is usually preserved in multiple system atrophy, possibly due to less cortical involvement in MSA.^10^ Most patients become wheelchair‐dependent within 5 years and die in the first 10 years after disease onset, showing dramatic disease progression.^11^

Here, we present a case of a person with MSA who presented with progressive loss of balance, gait abnormality, and combinations of autonomic failures; he was diagnosed with imaging and clinical criteria after 4 years of symptom onset, showing the diagnostic challenge in resource‐limited settings such as ours.

## 2 CASE HISTORY/EXAMINATION

A 55‐year‐old Black male individual presented to our neurology clinic with progressive loss of balance, gait abnormality, slurred speech, urinary urgency, nocturia, incontinence, loss of morning penile erection, and rigidity. These symptoms started 4 years ago; however, in the past 3 months, he developed choking episodes, intermittent hiccups, difficulty speaking at a regular pace, and slurred speech, signifying the recent progression of the disease. He visited multiple healthcare facilities and took levodopa/carbidopa 250/50 mg twice a day without significant improvement; rather, developing substantial side effects, such as irritability, anger, and aggressiveness, which prevented an increase in the dose. The patient is a married farmer with four children who completed his education at high school.

Physical examination revealed a body mass index (BMI) of 23.5 kg/m^2^, blood pressure of 130/80 mmHg (both in sitting and standing positions), heart rate of 92 beats/min, regular (both in sitting and standing positions), and respiratory rate of 16 breaths/min, with a regular breathing pattern. Neurologic examination revealed motor power of 4+/5 in the left upper extremity muscle groups; power in other muscle groups was normal; exaggerated deep tendon reflexes with clonus at the bilateral knee and ankle with upgoing plantar reflex. He also had cogwheel‐type rigidity in the left upper extremity and intention tremor in both hands. Cerebellar coordination abnormality was also observed as he could not properly perform finger‐to‐nose, heel‐to‐shin, and tandem walk tests. Cerebellar gait disturbance was observed with impaired gait velocity, stride length, slow swing speed, and stance time. The results of cranial nerve examination and other systemic examinations were unremarkable.

### 2.1 Methods

**FIGURE 1:**
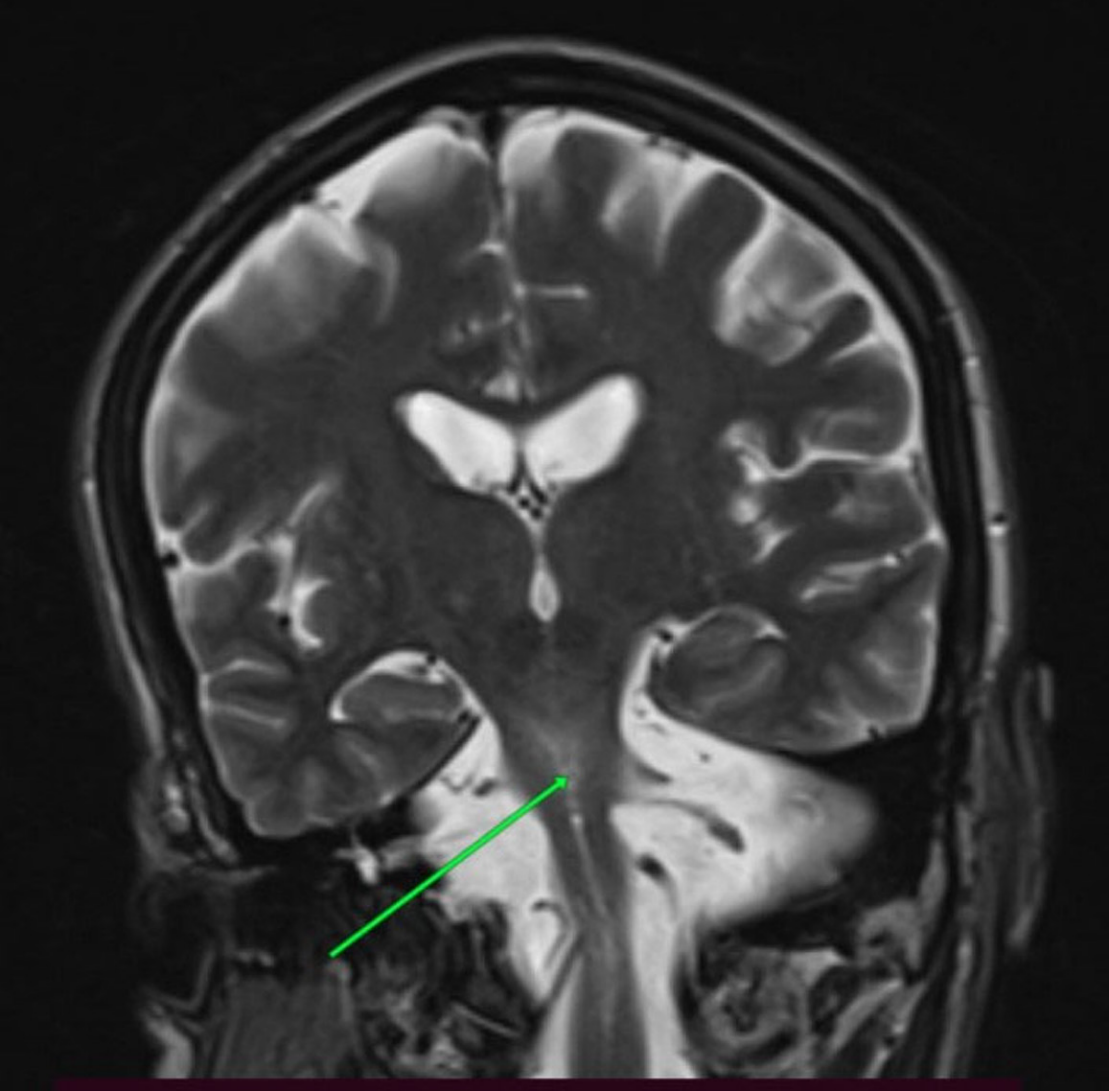
Pontine degeneration on T2‐weighted MRI(green arrow).

**FIGURE 2:**
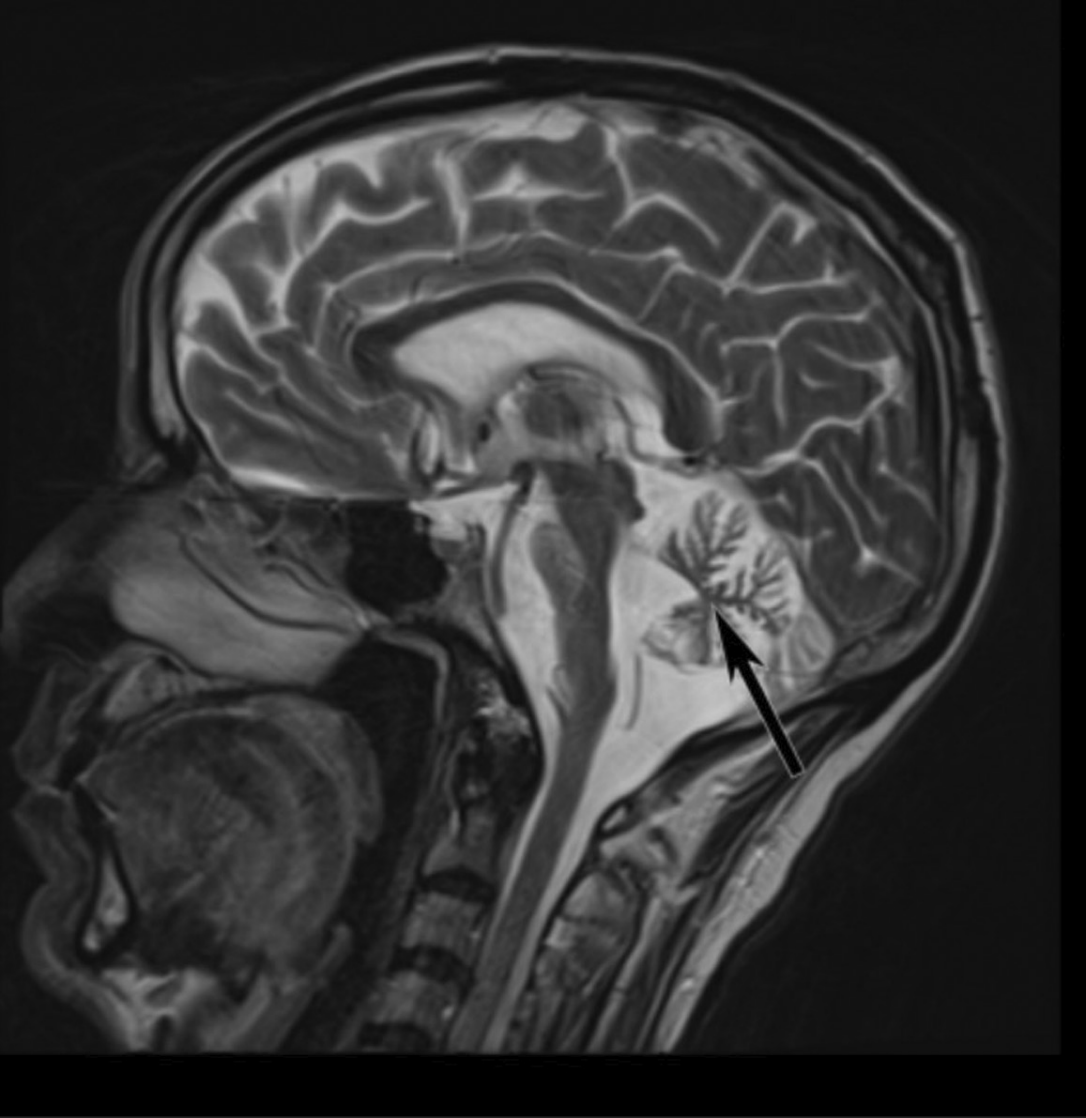
Cerebellar degeneration on T2‐weighted MRI (black arrow).

**FIGURE 3:**
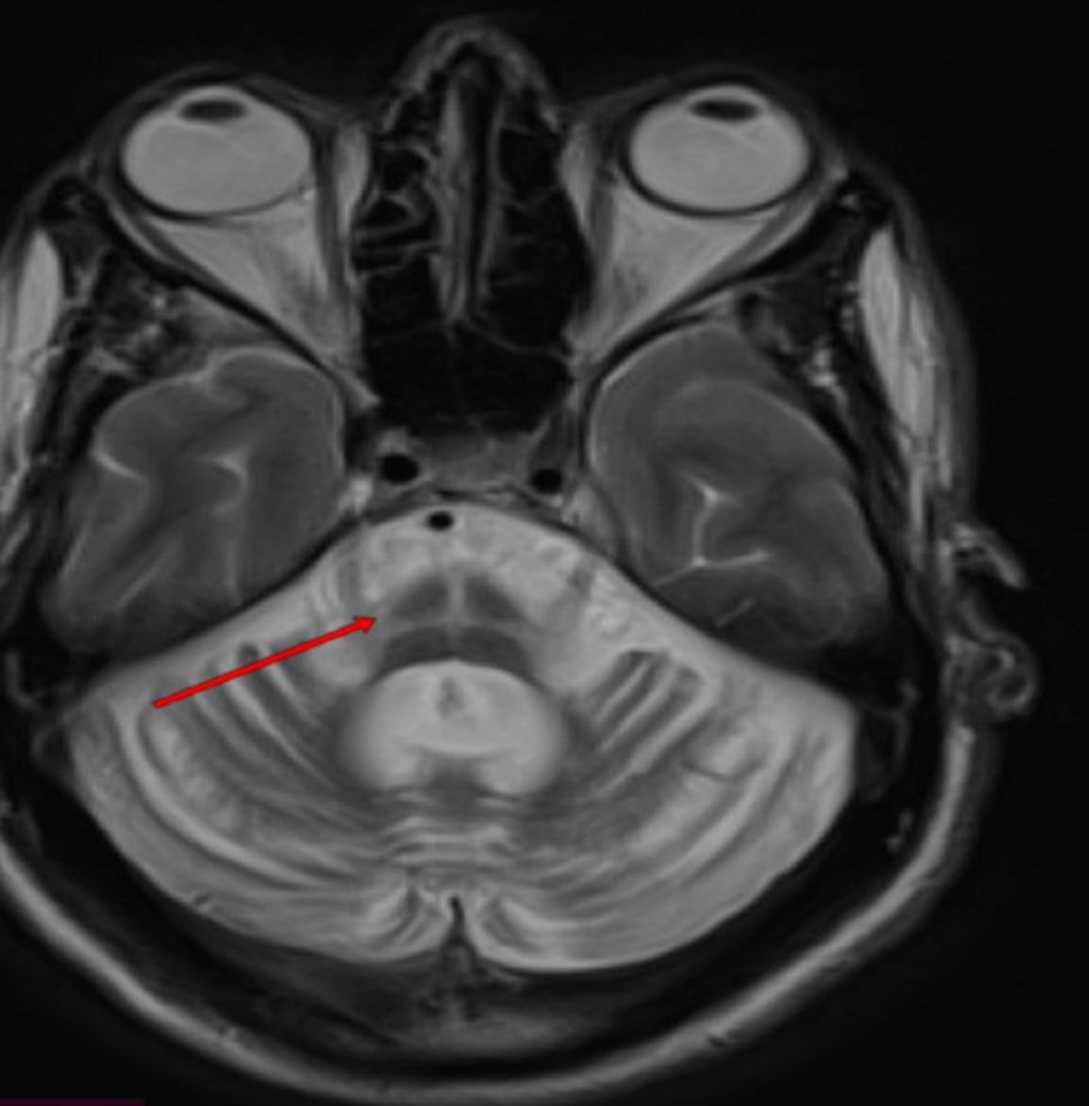
Axial T2‐weighted images showed the “hot cross bun” sign of the pons. (Red arrow).

Brain MRI showed marked pontocerebellar atrophy with a “hot cross bun” sign suggestive of the neurodegenerative process (Figures 1, 2, 3). Other laboratory investigations were within normal ranges, including complete blood count, renal function test, liver enzymes, fasting blood sugar levels, and serum electrolytes (Na+, K+, Mg + 2, Ca + 2, Cl‐ phosphate). Serological tests for HBSAG, HCV‐AB, VDRL, and PICT were also negative. His serum vitamin B12 level was within the normal range (705.3 pg/mL), and thyroid function tests were also normal (TSH‐1.3 IU/mL, free T4‐21.1 ng/dL, free T3‐2.28 pg/mL) (Table 1).

**TABLE 1:** Laboratory profile of the patient with normal reference ranges.

laboratory	Value	Reference range
WBC	5200 cells/μL	5000–11,000 cells/μL
Hemoglobin	14 mg/dL	13–16 mg/dL
Platelets	270,000 cells/μL	150,000–450,000 cells/μL
Creatinine	0.8 mg/dL	0.8–1.1 mg/dL
BUN	6 mg/dL	5‐20 mg/dL
AST	16 IU/L	5–35 IU/L
ALT	10 IU/L	5–35 IU/L
ALP	80 IU/L	50–180 IU/L
FBS	95 mg/dL	70–100 mg/dL
Serum sodium	138 mEq/L	135–145 mEq/L
Serum potassium	3.8 mEq/L	3.5–5.5 mEq/L
Serum magnesium	1.8 mg/dL	1.6–2.5 mg/dL
Serum calcium	8.4 mg/dL	8‐10 mg/dL
Serum chloride	102 mEq/L	95–105 mEq/L
Serum phosphate	3.5 mg/dL	3.4–4.5 mg/dL
Serum vitamin B12	705.3 pg/mL	>200 pg/mL
Serum TSH	1.3 IU/ml	0.3–5.6 IU/ml
Serum FT4	21.1 ng/dL	0.6–22 ng/dL
Serum FT3	2.28 pg/ml)	2.3–6.7 pg/mL
HBSAG	Negative	Negative
HCV‐Antibody	Negative	Negative
HIV test	Negative	Negative
VDRL	Negative	Negative

Abbreviations: ALP, alkaline phosphatase‐fasting blood sugar; ALT, alanine transaminase; AST, aspartate transaminase; BUN, blood urea nitrogen; FT3, free triiodothyronine; FT4, free thyroxine; HBSAG, hepatitis B surface antigen; HCV, hepatitis C virus; TSH, thyroid stimulating hormone; VDRL, venereal disease research laboratory; WBC, white bood cells.

He started physiotherapy for balance and strength along with speech therapy. Education and counseling regarding intermittent catheterization were also provided. Otherwise, he is not taking any drugs.

## 3 CONCLUSION AND RESULTS

This case report describes a 55‐year‐old man with multiple system atrophy cerebellar type who faced a diagnostic challenge due to resource limitations. The lack of access to advanced diagnostic tools like seed amplification assays and biomarkers resulted in a four‐year delay in diagnosis. The authors recommend wider availability of these tests for earlier and more accurate diagnosis of MSA. We also suggest performing a levodopa challenge test and implementing treatments focused on improving the patient's quality of life. The patient is still attending speech therapy and physiotherapy, but no significant improvement.

### 3.1 Discussion

Multiple system atrophy is an atypical Parkinson's plus syndrome involving the pyramidal nigrostriatal, autonomic, and cerebellar systems. It is a multisystem, sporadic disease with an unknown etiology characterized by different degrees of four cardinal clinical features, such as parkinsonism, cerebellar dysfunction, autonomic failure, and pyramidal signs.

The disease is also characterized by poor response to levodopa.^1^ Cell loss in the striatonigral and olivopontocerebellar regions of the brain and spinal cord, along with many characteristic glial cytoplasmic inclusions (GCIs) composed of fibrillated alpha‐synuclein proteins (referred to as primary alpha‐ alpha‐synucleinopathy), are neuropathological hallmarks of MSA.

There are two primary forms of multiple system atrophy: parkinsonian and cerebellar (MSA‐P and MSA‐C, respectively). Although the two varieties are quite similar, their primary distinction is the patient's symptoms at the initial diagnosis.^10^

Compared to MSA‐C, MSA‐P is more prevalent and is distinguished by the following symptoms: stiff muscles, tremors, bradykinesia, trouble bending limbs, and issues with balance and posture.^10^ In patients with MSA‐C, some individuals have difficulty swallowing or chewing.^10^ Despite their distinct features, MSA‐P and MSA‐C share symptoms of autonomic dysfunction, including erectile dysfunction, uncontrolled excessive or absent sweating, orthostatic hypotension, urinary dysfunction, and, in certain cases, psychological illnesses.^2^, ^10^

MSA is difficult to diagnose, and most patients are diagnosed with Parkinson's disease early in the clinical course. MSA diagnosis is based on clinical features. Definitive diagnosis can only be made through postmortem histological examination. High‐density glial cytoplasmic inclusions (GCIs) associated with degenerative changes in the nigrostriatal and olivopontocerebellar tracts confirm MSA.^12^, ^13^ MRI scans using T1 and T2 weighting are valuable tools for diagnosing MSA. These scans can reveal characteristic patterns, most notably the “hot cross bun” sign.^2^ Other imaging findings include the putaminal rim sign, atrophy of the cerebellum, and brain stem structures.^1^ MRI showed marked atrophy of the cerebellar peduncles, pons, and cerebellum, with the typical hot‐cross bun sign in the pons. Since our patient predominantly presented with cerebellar features with autonomic nervous system dysfunction together with typical MRI features, a probable diagnosis of MSA‐C was considered.

Seed amplification assays such as real‐time quaking‐induced conversion (RT‐QUIC) and protein misfolding cyclic amplification (PMCA) are becoming more popular in diagnosing MSA.^3^ However, the unavailability of these assays in resource‐limited areas such as ours makes the diagnosis of MSA challenging. Delay in diagnosis has negative impacts on the person with MSA, such as emotional distress, feelings of anxiety, and uncertainty about their diagnosis.^4^ Biomarkers in speculative CNS‐enriched extracellular vesicles are also evolving. They are promising for differentiating synucleinopathies from non‐synucleinopathies. Still, they are less reliable and highly heterogeneous for diagnosing MSA.^4^ Due to unavailability and financial reasons, we could not do our patient's seed amplification assays and biomarkers. This limitation contributed to the delay in diagnosis.

According to a study by Watanabe et al., the typical durations were 3, 5, 8, and 9 years from the start of symptoms to the point at which a person needed assistance to walk, use a wheelchair, remain bedridden, or pass away, respectively.^11^ Studies have shown that the majority of patients with MSA die from secondary complications of the disease, including aspiration pneumonia and pulmonary embolism.^11^ The cause of multiple system atrophy (MSA) remains unknown, and no current therapy can reverse or halt disease progression. Most of the therapeutic options include symptomatic treatment. For approximately one‐third of patients with MSA, L‐dopa is a successful treatment for Parkinsonism symptoms; for MSA‐C, physical therapy is the best course of action.

When treating patients' symptoms, the goal should be to address issues that lower their quality of life, such as depression, poor self‐control, and motor impairment.^14^

We report the case of a 55‐year‐old man with MSA‐C who was diagnosed after 4 years with typical symptoms and signs with supportive evidence of MRI features, according to the second consensus on the diagnosis of MSA. Limited access to advanced diagnostic tools, such as seed amplification assays and biomarkers, likely delayed the accurate diagnosis. We recommend making these tests more widely available to improve early detection of MSA and similar conditions. Additionally, a levodopa challenge test should have been performed on this patient.

Although MSA does not have treatment, pharmacological and non‐pharmacological therapies for symptom control and improving quality of life are recommended.

## AUTHOR CONTRIBUTIONS

**Gebeyehu Tessema Azibte:** Software; writing – review and editing. **Bereket Abraha Molla:** Writing – original draft. **Sebhatleab Teju Mulate:** Resources; writing – review and editing. **Selam Kifelew Melkamu:** Supervision; validation. **Zekarias Seifu Ayalew:** Writing – review and editing.

## FUNDING INFORMATION

We received no financial support for this case report.

## CONFLICT OF INTEREST STATEMENT

We declare that there are no competing interests.

## ETHICS STATEMENT

There are no ethical concerns about this case report.

## CONSENT

Written informed consent was obtained from the patient to publish this report in accordance with the journal's patient consent policy.

## Data Availability

Data openly available in a public repository that issues datasets with DOIs.
